# The core competencies of a health education teacher

**DOI:** 10.1093/heapro/daae078

**Published:** 2024-07-10

**Authors:** Olli Paakkari, Markus Kulmala, Nelli Lyyra, Terhi Saaranen, Pirjo Lindfors, Heli Tyrväinen

**Affiliations:** Faculty of Sport and Health Sciences, Research Centre for Health Promotion, University of Jyväskylä, Keskussairaalantie 4, 40014, Finland; Faculty of Sport and Health Sciences, Research Centre for Health Promotion, University of Jyväskylä, Keskussairaalantie 4, 40014, Finland; Faculty of Sport and Health Sciences, Research Centre for Health Promotion, University of Jyväskylä, Keskussairaalantie 4, 40014, Finland; Faculty of Health Sciences, Department of Nursing Science, University of Eastern Finland, Yliopistonrinne 3, 70211 Kuopio, Finland; Faculty of Social Sciences, Unit of Health Sciences, Tampere University, Arvo Ylpön katu 34, 33014, Finland; Health Sciences, Open University, University of Jyväskylä, Alvar Aallon katu 9, 40014, Finland

**Keywords:** competence, health education, teacher, teacher education, school

## Abstract

Teachers play a crucial role in students’ learning and in the development of health literacy. Hence, the aim of this study was to identify the core competencies needed for teachers of health education in supporting student learning. A three-round Delphi study was carried out over an 8-week period, through consultation with 25 Finnish experts in health education. An open-ended question was used to identify the core competencies for school health educators. The data were analysed using inductive content analysis. In subsequent rounds, experts were asked to assess the importance of the identified competencies on a 7-point Likert scale, and finally to rank the most important competencies. In total, 52 competencies were identified and categorized into eight core competence domains. Thereafter, 40 competencies were assessed and selected for the third round, in which the experts ranked the 15 most important competencies, encompassing four core domains, i.e. pedagogic and subject-specific didactic, social and emotional, content knowledge and continuous professional development. Other domains of competence identified in the present study were ethical competence, competence in school health promotion, contextual competence and professional well-being competence. The study defines health education teacher core competencies and domains, and the information can be used in teacher education programmes, for developing teaching and for teachers’ self-evaluation.

**Contribution to Health Promotion**

- Competent health education teachers can have a significant impact on students’ health literacy.
- This study identifies the extensive list of competencies needed for teachers of health education.
- The results of the study can be used to build competence-based curricula and guide the development of future health education teacher education programmes.

## BACKGROUND

Teaching is a complex and demanding profession that requires a wide range of expertise. Teachers form one of the key factors in students’ learning and academic achievement (Hattie, 2009), but there are differences between teachers (Stronge *et al.*, 2007; Hattie, 2009). Competent teachers influence the quality of teaching and thus student learning, and various explanations of these chains of effects have been offered (Fauth *et al.*, 2019; Blömeke *et al.*, 2022). The importance of teachers’ work for students’ learning and development places high demands on teachers’ initial training and on continuous professional development. From the perspective of health education (HE) teacher training, it is critical to take a holistic view of the teacher’s work and to consider the competencies that teachers need to succeed in their profession. This broad understanding of the teacher’s profession is fundamental, and relevant factors can also support teachers in constructing strong professional identities. There is thus a need for a conceptually coherent framework of teacher competencies, based on knowledge of teaching and learning, and taking into account the complexity of the teacher’s work (Grossmann and McDonald, 2008).

One key aspect relevant to identifying HE teacher competencies is that the school reaches almost the whole age group at any given time. It thus offers an excellent opportunity to develop health literacy, regardless of the individual’s background (Paakkari and Paakkari, 2012). Health literacy has been found to be a constitutive determinant for health across age groups (Berkman *et al.*, 2011; van der Heide *et al.*, 2016; Paakkari *et al.*, 2020), and low health literacy has been identified as an independent risk factor (Volandes and Paasche-Orlow, 2007). Among children and adolescents, health literacy has emerged as an independent factor explaining health disparities, with higher health literacy being related to more positive health outcomes (Paakkari *et al.*, 2019a). Thus, competent HE teachers have the possibilities to benefit a nation’s health, while providing quality education for future adults (St Leger and Nutbeam, 2000).

Internationally, HE in schools is organized in diverse ways. HE can be an independent and obligatory school subject taught by teachers with a degree in the subject, or health topics may be integrated within other school subjects. Regardless of how HE is organized, it is essential for the development of the subject and for students’ learning that teachers have sufficient competence. A conceptually coherent competence framework can provide a basis for relevant and versatile teachers’ competencies, allowing them to perform well in different contexts.

The concept of competence is characterized by controversy, ambiguity and contradiction (Schneider, 2019), and there is variation in key constructs and domains, depending on scientific and academic disciplines and education, and policy cultures across countries (Caena, 2014). Competence can be defined as the capability to perform tasks, as a learnable and contextualized disposition, as a process, as a relation between abilities and the completion of a task, as a quality or state of being or as a behaviour integrating resources (Schneider, 2019). It is a combination of attributes such as knowledge, skills, dispositions and attitudes (Hager and Gonczi, 1996; European Commission, 2013; Blömeke *et al.*, 2015) which construct the ability to successfully perform domain (subject) task-specific actions (Blömeke *et al.*, 2015; Schneider, 2019). In this article, the concept encompasses the professional demands related to the functions, responsibilities and roles of the HE teacher in the subject-specific context.

The literature on the core competencies of teachers contains studies examining the general competencies needed in the work of a teacher, as well as studies from the perspective of more specific contextual requirements. Topic- or subject-specific competencies have been defined for teachers in various domains, including sustainable development (Lohman *et al.,* 2021), digital competence (European Commission *et al*., 2017), collaborative learning (Kaendler *et al.*, 2015), sexuality education (World Health Organization, 2017) and science (Nouri *et al*., 2021).

An interest in holistic, dynamic and process-oriented approaches has increased within research on teacher competencies (Caena, 2014; Metsäpelto *et al.*, 2021). Competence development processes and transformation into performance can be viewed as personally, situationally and socially determined (Blömeke and Kaiser, 2017). All higher education graduates need generic competencies, such as conceptual skills (e.g. problem-solving, thinking skills, creativity, information processing), social skills (e.g. communication, teamwork, leadership) and personal skills (e.g. lifelong learning, critical reflection, social responsibility) (Strijbos *et al.*, 2015). It has been suggested that the competencies common to all teachers include well-structured knowledge of education theories and curricula, solid knowledge on how to teach specific subjects, classroom management strategies and skills, reflective and research skills (including a commitment to professional development), collaborative skills and the ability to adapt to different situations within schools (European Commission, 2013; Caena, 2014).

On the basis of previous studies (e.g. Shulman, 1987; Baumert and Kunter, 2013; Blömeke *et al.*, 2015; Blömeke and Kaiser, 2017; Klassen and Kim, 2019), Metsäpelto *et al.* (2021) constructed a multidimensional adapted process model of teaching (MAP model); this identifies the relevant competence domains and is applicable to teachers in a wide range of teaching professions and school subjects. The MAP model refined the competencies of Blömeke *et al.*’s (2015) model, framing them as observable teaching practices. The MAP model emphasizes the situation-specific skills of perceiving, interpreting and making decisions, teaching and professional practices as indicators of teaching competencies. In addition, the MAP model contains a set of individual competencies. These consist of possession of the knowledge base, cognitive thinking skills, social skills, personal orientations and professional well-being (Metsäpelto *et al.*, 2021).

There has been relatively little research on the specific competencies of HE teachers. The empirical study of Moynihan *et al.* (2015) aimed to identify HE teachers’ core competencies in supporting the development of health literacy. The study identified 12 competencies, divided into three overlapping competence domains: knowledge (about curricula, health determinants, learners, HE theories and models, general pedagogical knowledge and pedagogical content knowledge), skills (communication, ethical thinking, researching, planning and implementing initiatives) and attitude. Similarly, the theoretical framework of Szucs *et al.* (2021) describes knowledge and skills as core competence domains for teachers delivering HE; however, the third competence domain is that of personal characteristics (e.g. an academic degree in HE, confidence, beliefs, cultural responsiveness and humility, a sense of equity). The knowledge domain encompasses five categories (learner characteristics and development, pedagogical knowledge, subject content knowledge, professional standards), while the essential skills domain includes learning environments, content and delivery and collaboration and learning (Szucs *et al.*, 2021). In addition to these studies, there are some national (USA) guidance documents aiming to standardize HE teacher competencies (National Board for Professional Teaching Standards, 2002; Society for Health and Physical Educators, 2018).

Based on the paucity of previous empirical studies and the fact that HE teachers’ voices were not taken into account in the studies in question, the present study aimed to identify and describe competencies relevant to HE teachers’ work. It is envisaged that the results of the study could be used in the development of HE teacher education.

## METHODS

A Delphi approach was utilized in this study, with three survey questionnaire rounds over an 8-week period in 2022. The Delphi method has been considered appropriate in cases where research-based knowledge of the topic is scarce (Jünger *et al.*, 2017). In this method, in which both qualitative and quantitative processes are used, selected expert panel members give individual opinions, the aim being to generate a consensus opinion based on these views (Nasa *et al.*, 2021). The key principles of the process are anonymity, an iterative questionnaire procedure and controlled feedback (Jünger *et al.*, 2017; Staykova, 2019; Nasa *et al.*, 2021). The rationale for anonymous responses is the avoidance of social pressure that would produce conformity to a dominant view (Jünger *et al.*, 2017). In a similar vein, there were no feedback discussions in the present study, since these can affect individuals’ responses and create a biased consensus (Barrios *et al.*, 2021). After each survey round, the data collected were analysed and presented to the expert panel in the form of another questionnaire, presented in the next round.

In the present study, the Delphi rounds were conducted via online questionnaires, with the anonymity of the responses secured. The questionnaire for each round was pre-tested by external researchers and by teachers with experience in the field of the study. Based on their comments, minor changes were made to the questionnaires. The participants received general information on the process of the study via email between the rounds.

### Participants

One of the most important steps in a Delphi study is the selection of the panel members, i.e. experts (Green *et al.*, 1999). Adequate heterogeneity of panel members helps to provide a broader picture of the phenomenon under study. There is no precise definition of the size of an expert panel; in fact, panels typically range from 10 to 100 members, and in the health sciences, 20–50 members are deemed sufficient for a Delphi study (Niederberger and Spranger, 2020; Nasa *et al.*, 2021). What matters in a Delphi study is the expertise and representativeness of the experts, rather than the number of panellists (Staykova, 2019). Bearing in mind the complexity of the phenomenon under study (teacher competencies), the study aimed to achieve a comprehensive picture of the subject. A sufficiently large number of experts from different backgrounds can increase the diversity of the responses and the possibilities to generalize results (Nasa *et al.*, 2021). For these reasons, this study aimed at a panel size greater than the minimum number, i.e. a medium double-digit range typically used in Delphi studies (Diamond *et al.*, 2014; Niederberger and Spranger, 2020; Nasa *et al.*, 2021). The selection of experts was based on pre-defined criteria (Jünger *et al.*, 2017; Nasa *et al.*, 2021). These criteria included appropriate education and an academic degree in HE, relevant expertise with long experience in the subject and an active role in the development of HE. A pre-selected list of 29 HE experts was formulated based on the criteria, and these experts were invited to participate in the study. All the experts were contacted personally to explain the purpose and method of the study. In total, 25 experts (all from Finland) agreed to participate in the study (Table 1).

**Table 1:** Participants’ demographic data by round

		Round 1 *n* = 25 *n* (%)	Round 2 *n* = 23 *n* (%)	Round 3 *n* = 24 *n* (%)
Gender	Male	6 (24)	6 (26)	5 (22)
	Female	19 (76)	16 (70)	19 (78)
	Other	0 (0)	0 (0)	0 (0)
	Prefer not to disclose	0 (0)	1 (4)	0 (0)
Years of work in health education	Mean	19.7	19.5	18.6
	Median	20	20	20
	SD	6.4	7.3	6.2
Highest degree obtained	Master’s	16 (64)	13 (57)	14 (61)
	PhD	9 (36)	10 (43)	10 (39)
Pedagogical studies for teachers (60 ECTS)	Yes	25 (100)	23 (100)	24 (100)
	No	0 (0)	0 (0)	(0)
Professional title	Lecturer in comprehensive school, upper secondary school or vocational education	10 (40)	7 (30)	8 (30)
	Lecturer in teacher education or university teacher	10 (40)	13 (57)	12 (52)
	Professor	2 (8)	2 (9)	2 (9)
	Other	3 (12)	1 (4)	2 (9)

In aiming to define the teacher’s competencies, it is important to take into account the authentic challenges of a HE teacher’s work. In this study, the panellists included experienced teachers and teacher trainers with an understanding of the different aspects of a teacher’s work. This was done to ensure that the study would be maximally applicable to HE teacher education and teacher competence development.

Invitations were issued to teacher trainers from each of the Finnish universities providing teacher training in HE, and to experienced HE teachers working at different school levels. The experts had diverse educational backgrounds and competence in areas such as teacher education, health promotion, management, health organizations and research. The panellists included people who had been active in the development of teaching; also those who had built the theoretical basis of the subject, been involved in the matriculation examination board, produced learning materials and HE textbooks, developed national and local curricula and reformed assessments.

### Delphi procedure

In the first round, the experts were given the opportunity to freely detail competencies they deemed relevant to a HE teacher’s work. Open-ended questions were framed: ‘What do you consider to be relevant competencies in HE teachers’ work? Name and describe in detail as many competencies as possible’. The study researchers performed inductive content analysis on the answers (Kyngäs, 2020). As a first step, the data (i.e. the experts’ responses) were carefully reviewed and read through multiple times by the researchers. Some of the longer expressions were slightly condensed, while ensuring that the idea still corresponded to the raw data. Thereafter, individual similar or identical original expressions were combined, leaving a comprehensive list of all the competencies mentioned in the experts’ answers. Finally, content similarities and differences were compared to determine which competencies could be grouped together. The main categories emerged from the shared content of the group. The researchers named the categories, applying their expertise and theoretical understanding. Any disagreements or discrepancies were resolved by open discussion to reach a final consensus. The experts’ expressions were followed closely in constructing the items for the second-round questionnaire.

In the second round, experts were asked to evaluate the importance of the competencies identified in the first round, using a 7-point Likert scale. The scale ran from 1 = not at all important to 7 = very important. The most important competencies for inclusion in the third-round questionnaire were selected, applying four criteria and pre-defined cut-off values (Nasa *et al.*, 2021) as follows: 5 for the median, 2 for the interquartile ranges (IQRs), 60% for the proportion of respondents who gave a rating of at least 6 on the Likert scale, and 22% for pairwise agreement (twice the agreement compared to a situation in which the respondents rated the item as maximally different). The pairwise agreement was calculated by dividing the number of agreeing pairs of raters by the number of all possible pairs in the dataset.

According to systematic reviews, in Delphi studies a consensus is commonly defined on the basis of the percentage of agreement, the central tendency (the median in this study), or a combination of these (Diamond *et al.*, 2014; Jünger *et al.*, 2017). However, the definition of an appropriate agreement percentage varies widely and is to some extent arbitrary, given that there are no clear guidelines or commonly accepted criteria for determining a consensus (Diamond *et al.*, 2014; Jünger *et al.*, 2017; Nasa *et al.*, 2021). In addition to the values typically used to describe a consensus (percentage of agreement, central tendency), two other values were used in this study. These were intended to provide more diverse information on the variation between respondents, and in this way provide more evidence that an appropriate consensus had been reached.

In setting cut-off values for the study, the aim was to ensure that the key issues were reliably selected from the data. Hence, cut-off values of 2 for the IQR and 22% for pairwise agreement were chosen, so that the pool of competencies would be limited to those displaying less variability between raters. The IQR range was preferred to the raw first and third quartiles, since it captures the amount of variability more concisely. To ensure that all the competencies were deemed highly important, it was decided that, for any given competency, at least 60% of the respondents should give it a rating of at least 6 on the Likert scale. These values were based on the best judgement of the research group, because there is no clear research-based guidance on the exact threshold values or on the range of these values.

In the third round, the experts were asked to select and rank the top 15 competencies from the list generated in the second round. The most important item received the highest value, i.e. 15, and the least important item the value of 1. Items outside the list of the 15 most important items received a value of 0. Based on this, a rank sum was calculated. Agreement among the experts was examined via the pairwise agreement, the proportion of experts who ranked a certain competence as being among the 15 most important items, and Kendall’s concordance coefficient *W*; the latter measures the agreement among raters, and takes values between 0 and 1, with 1 representing total agreement between raters. The applied critical values (although by nature arbitrary) were, as presented in Landis and Koch (1977), as follows: for Kendall’s concordance, a coefficient *W* of 0.00 ≤ *W* < 0.20 indicates slight agreement, 0.20 ≤ *W* < 0.40 fair agreement, 0.40 ≤ *W* < 0.60 moderate agreement, 0.60 ≤ *W* < 0.80 substantial agreement and *W* ≥ 0.80 almost perfect agreement. The pairwise agreement was calculated by first categorizing the data into groups composed of five ratings plus the group of 0 (0, 1–5, 6–10, 11–15) and then dividing the number of agreeing pairs by the number of all possible pairs in the dataset.

The present Delphi study consisted of three rounds, as described above. Traditionally, a Delphi study is thought to require at least four rounds (Nasa *et al.*, 2021), but systematic reviews have shown that the majority of studies have used either two or three rounds, with the number of rounds varying from one to five (Diamond *et al.*, 2014; Jünger *et al.*, 2017). On this basis, it was anticipated that the current study would involve three to four rounds, depending on the degree of consensus achieved. Controlled feedback, presented to the expert panel in the form of another questionnaire in the next round, could support consensus building and thus reduce the number of rounds needed. When the pre-defined criteria with the chosen cut-off values were met, it was concluded that three rounds were sufficient to achieve a reliable result.

### Ethics

This study followed the ethical principles of research with human participants (Finnish National Board on Research Integrity, 2019), and the research was approved by the institutional ethics committee. Active consent was obtained from all participants at each stage of data collection. Participants were informed about the purpose of the study, its design, the anonymous response and the voluntary nature of participation. Data privacy was implemented appropriately.

## RESULTS

### First and second round

In the first Delphi round, experts produced in total 343 expressions describing competencies. Using content analysis, 52 competencies were rephrased from the data as a sentence with concrete examples, and categorized into eight core competence domains (Table 2). The largest domain (*n* = 19) was *Pedagogic and subject-specific didactic competence*; this refers to classroom management, planning, instruction, assessment (in general and from a subject-specific perspective), plus understanding the cognitive, motivational and emotional factors that regulate students learning. The second largest domain, *Social and emotional competence* (*n* = 9) refers to the teacher’s ability to relate to other people; it includes being aware of one’s own and others’ emotions, regulating a supportive emotional atmosphere, and respecting diversity. The domain of *Content knowledge* (*n* = 3) encompasses teachers’ knowledge of key concepts, facts, theories and phenomena in the subject area, plus comprehension of the structure of the subject and how this knowledge is generated. *Ethical competence* (*n* = 5) emphasizes the teachers’ ability to commit to professional ethics, to assess and justify their work from an ethical perspective, to solve ethical problems in the school and to reflect on their own values, attitudes and principles, including the consequences ensuing from them.

**Table 2:** Core competence domains and a list of competencies based on content analysis from the first Delphi round, arranged within the domains according to the consensus criteria used in the second Delphi round (median, IQR with first and third quartiles, pairwise agreement and proportion with value 6 on a Likert scale 1–7)

Core competence domain	Competencies	Median	Proportion % ≥ 6	Q_1_	Q_3_	IQR	Pairwise agreement
Pedagogic and subject-specific didactic competence	Ability to look at the teaching-learning process of health education comprehensively (planning-implementation-assessment and impact of choices made at these stages on each other)	7	100	6	7	1	48
	Ability to design logically-progressing educational modules and individual lessons according to the curriculum (health literacy, objectives, content), taking into account students and groups	7	95.7	7	7	0	63
	Ability to plan motivating and meaningful learning situations and to support learners’ self-efficacy	7	95.7	6.5	7	0.5	58
	Group management skills	7	95.7	6	7	1	45
	Skill to give feedback to the student	7	91.3	6	7	1	45
	Assessment competence (e.g. skills to design and implement criteria-based and ethically sustainable diagnostic, formative and summative evaluation, targeting of evaluation)	7	91.3	6	7	1	45
	Situation-specific competence in lessons (e.g. observation, interpretation, decision-making in learning, flexibility, ability to change activities in a teaching situation if appropriate)	7	87.0	6	7	1	43
	Ability to take into account the specific characteristics of the subject in learning situations (e.g. the personal nature of the contents, cultural ties, sensitivity)	7	87.0	6	7	1	40
	Teaching skills, encompassing the ability to use various learning environments (including authentic and digital environments), working methods, and learning materials; also the ability to promote the active participation of the learner and to demonstrate matters as required	7	82.6	6	7	1	42
	Ability to develop students’ thinking skills (e.g. versatile information-processing)	7	78.3	6	7	1	37
	Knowledge of students and groups (e.g. health behaviour, growth and development, youth cultures, background/growth environment, prior knowledge of the subject to be taught, concerns), plus the ability to strengthen student and group knowledge	7	73.9	5.5	7	1.5	36
	Knowledge of the general and subject-specific parts of the curriculum	6	82.6	6	7	1	33
	Knowledge of cognitive, emotional and motivational factors governing the learning of students and obstacles to learning	6	78.3	6	7	1	30
	Ability and willingness to differentiate teaching according to the needs of the student and the group	6	73.9	5.5	7	1.5	29
	Knowledge of general pedagogical principles and theories related to teaching and learning	6	73.9	5.5	7	1.5	29
	Ability to guide students in study skills, and to support them in setting goals and evaluating their achievement	6	65.2	5	7	2	31
	Ability to plan and develop a local curriculum on the basis of the national curriculum	6	60.9	5	6.5	1.5	23
	Organizational and classroom management skills (e.g. organizing class activities)	6	60.9	5	7	2	25
	Digital skills, ICT skills [Removed Round 3]	6	56.5	5	6	1	34
Social and emotional competence	Ability to create a safe learning environment	7	100	6.5	7	0.5	60
	Interactional competence (e.g. active listening, asking, guiding interaction situations and conversation, genuine presence and encounter, encouragement of discussion)	7	100	6	7	1	56
	Emotional competence (e.g. identification and regulation of the teacher’s own feelings, putting oneself in the position of another person, i.e. empathy)	6	91.3	6	7	1	40
	Communication skills (e.g. readiness for spoken and written communication, informing, preparing instructions, readiness to communicate sensitively)	6	87.0	6	6	0	47
	Ability to take into account diversity and to act in a multicultural classroom and school community	6	82.6	6	7	1	35
	Cooperation competence (e.g. ability to work towards a common goal, co-operation with homes, colleagues, other school staff, other stakeholders)	6	73.9	5.5	7	1.5	28
	Leadership, taking and bearing responsibility in the school community and in teaching situations [Removed Round 3]	6	52.2	5	6	1	23
	Networking competence (e.g. ability to create networks, participation in a professional community, shared expertise) [Removed Round 3]	5	39.1	4	6	2	21
	International competence (e.g. language skills, ability to create international networks) [Removed Round 3]	4	13.0	3	4.5	1.5	19
Content knowledge	Content knowledge of health education (e.g. content, concepts, current issues and phenomena to be taught)	7	95.7	6	7	1	53
	Ability to understand the knowledge structure of the learning content of health education (expansion and deepening of the content from one school level to the next), core content, the connections between the content and the comprehensive phenomena arising from the content	7	91.3	6	7	1	52
	Ability to identify the diverse nature of knowledge related to the subject (e.g. multidisciplinarity, how knowledge is produced, who produces knowledge, changes in knowledge)	6	69.6	5	7	2	27
Ethical competence	Ability to commit to the ethical responsibility of the teacher’s work (e.g. truthfulness, justice, freedom and responsibility, dignity, reliability)	7	100	7	7	0	76
	Ability to guide students towards ethical thinking (e.g. building safe learning situations that include ethical reflection and argumentation)	7	95.7	6	7	1	44
	Ability to identify one’s own values, principles, attitudes and views, plus their significance for personal pedagogical and content choices	7	91.3	6	7	1	42
	Ability to analyse and solve ethical problems arising in the work of a health education teacher	7	91.3	6	7	1	40
	Ability to assess and justify the work of a health education teacher from an ethical point of view	6	69.6	5	7	2	26
Competence in school health promotion	Awareness of the role, importance and activities of the teacher as a health promoter for students (e.g. the ability to address the student’s concerns and to guide them, if necessary, to the right kind of help, promoting mental health in the classroom)	6	82.6	6	7	1	36
	Ability to plan, implement and evaluate initiatives/projects/programmes promoting community well-being and the health of the whole school, and to support the collective ability of staff to promote health [Removed Round 3]	5	39.1	4	6	2	18
	Ability to understand the overall health promotion of the school community (e.g. goals, participants, areas of responsibility, subjects, policies) [Removed Round 3]	5	30.4	5	6	1	34
	Administrative and financial competence (can plan and implement health-promoting activities from the perspectives of administration and finance) [Removed Round 3]	4	0	3	4	1	21
Contextual competence	Ability to understand the socio-cultural and social context of the health education subject (e.g. norms and values, political, cultural, historical and economic factors, taking into account local context factors such as school, residential area, families)	6	65.2	5	6.5	1.5	26
	Ability to study widely the starting points and objectives of the subject in relation to current and future challenges (e.g. the nature and ethos of the subject, planetary well-being, peace education, globalization, human rights, inequality, over-consumption, eco-health education, democracy education) [Removed Round 3]	5	47.8	5	6	1	26
	Knowledge of disciplines related to the health education subject, socially significant organizations and influence channels [Removed Round 3]	5	21.7	4	5	1	30
Continuous professional development competence	Ability to reflect, i.e. critical examination of one’s own thinking, competence, teaching and action (e.g. values, attitudes, emotions, motives, awareness of one’s own perception of learning, humanity and knowledge), plus readiness to change one’s own actions following reflection	7	100	6	7	1	53
	Ability and willingness to maintain and develop one’s own professional skills (e.g. collecting and utilizing feedback, innovativeness, learning skills, enthusiasm and motivation for development, self-directiveness)	7	87.0	6	7	1	51
	Ability to search for, structure and evaluate information	6	82.6	6	7	1	35
	Critical thinking and problem-solving skills (e.g. applying, analysing, evaluating, reasoning, justifying, and creating new knowledge), plus awareness and understanding of one’s own thinking processes (metacognition)	6	73.9	5.5	7	1.5	30
	Ability to examine the competence of the health education teacher in a broad and comprehensive manner (touching on the integrity of competencies, connections, and their impact on one another) [Removed Round 3]	6	69.6	4.5	7	2.5	28
	Evidence-based approach (use of effective practices and methods) [Removed Round 3]	5	47.8	5	6.5	1.5	25
	Research competence (management and application of the principles of scientific research in studying one´s own work) [Removed Round 3]	5	39.1	4	6	2	23
Professional well-being competence	Ability to maintain and promote one’s well-being at work (e.g. skills in recovery, stress management, limiting work, planning and managing time use)	7	91.3	6	7	1	41
	Resilience (e.g. flexibility, tolerance of incompleteness and uncertainty, readiness for change, readiness to recover from unexpected and difficult situations)	7	87	6	7	1	38

The domain *Competence in school health promotion* (*n* = 4) refers to the ability to understand school health promotion as a whole, plus the ability to plan, implement and evaluate school-wide health promotion projects or programmes. *Contextual competence* (*n =* 3) contains an understanding of the socio-cultural context in which teaching occurs. The *Continuous professional development competence* (*n* = 7) refers to the ability and willingness to maintain and develop one’s own professional expertise, and to incorporate new understandings within practice. The last domain, the *Professional well-being competence* (*n =* 2), includes the ability to maintain and promote one’s own well-being at work, and also teachers’ resilience as a dynamic interplay between protective and stress factors.

In the second Delphi round, 40 of the 52 original competencies were selected as the most important for HE teachers’ work based on four criteria mentioned above and their pre-defined cut-off values, thus establishing the consensus among the expert panellists (Table 2). Most of the competencies (*n* = 43) had a median value of 7 or 6, and the median for the remaining nine competencies was 5 or 4. The level of agreement between the experts was comparatively high for all the competencies, being highest for the most important items and slightly lower for items with a median of 6. The agreement was lowest among competencies with a median of 5 or 4, but still relatively strong.

All the competencies that fell under the domains of *Ethical competence* (*n* = 5), *Content knowledge* (*n* = 3) or *Professional well-being competence* (*n* = 2) were selected for the third Delphi round. In the largest competence domain, *Pedagogic and subject-specific didactic competence* (*n* = 19), only one competence was excluded. Three competencies were removed from each of the domains of *Social and emotional competence* (*n* = 9), *Competence in school health promotion* (*n* = 4) and *Continuous professional development competence* (*n* = 7). Two competencies were omitted from the *Contextual competence* (*n* = 3) domain.

### Third round

With 24 expert panellists in the final Delphi round, the theoretical maximum for the inverse sum score was 360 if all experts had chosen the same competence as the most important. The five most important competencies were content knowledge (score 211), teaching skills (score 204), interactional competence (score 187), the ability to reflect (score 155) and the ability to design logically progressing educational modules in line with the curriculum (score 149) (Table 3). At least three-quarters of the experts ranked these competencies in the top 15 most important. The sum score of the remaining 10 competencies varied between 87 and 129. Moreover, 46%–71% of the experts ranked these competencies in the top 15, with reasonably good pairwise agreement (26%–34%). Overall, the agreement between experts in the third round could be regarded as fair according to Kendall’s *W* (0.22; *Χ*²(39) = 205.86; *p* < 0.001).

**Table 3:** The most important HE teacher competencies (third Delphi round)

Competencies	Rank	Sum	Percentage in top 15	Pairwise agreement
Content knowledge of health education	1	211	75.0	35
Teaching skills, ability to use various learning environments, working methods and learning materials, ability to promote the active participation of the learner and to demonstrate matters	2	204	75.0	31
Interactional competence	3	187	75.0	28
Ability to reflect	4	155	83.3	23
Ability to design logically progressing educational modules and individual lessons	5	149	75.0	24
Assessment competence	6	129	70.8	32
Ability to create a safe learning environment	7	120	54.2	30
Ability to plan motivating and meaningful learning situations and to support learners’ self-efficacy	8	117	58.3	26
Ability to develop students’ thinking skills	9	107	45.8	37
Ability and willingness to maintain and develop one’s own professional skills	10	99	58.3	29
Knowledge of students and groups, plus the ability to strengthen student and group knowledge	11	93	50.0	31
Ability to guide students in study skills, and to support them in setting goals and evaluating their achievement	12	91	50.0	31
Ability to look at the teaching-learning process of health education comprehensively	13	89	45.8	34
Ability to understand the knowledge structure of the learning content of health education	14	88	54.2	29
Critical thinking and problem-solving skills, plus awareness and understanding of one’s own thinking processes (metacognition)	15	87	45.8	34

**Fig. 1:**
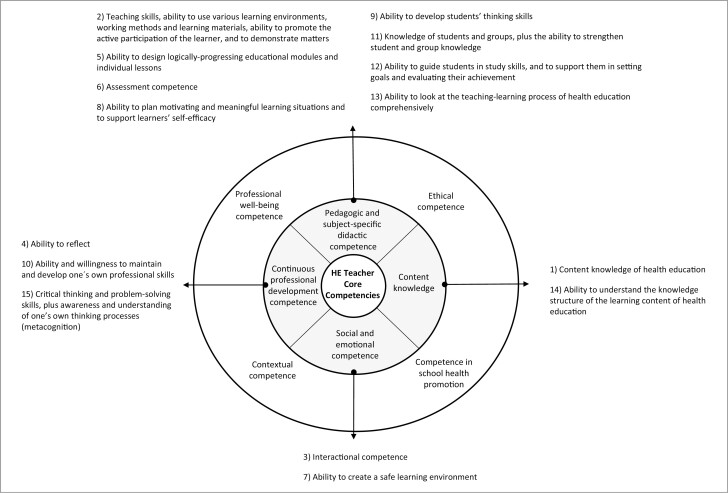
Health education teacher core competence domains and the related 15 most important competencies, numbered according to rank. Fundamental competencies are presented in gray, and supportive competencies are in the outer circle.

The distribution of the 15 most important competencies in terms of the core competence domains is shown in Figure 1. Most of the important competencies (*n* = 8) belonged to the domain of *Pedagogic and subject-specific didactic competence.* Other competence domains that received mentions were *Continuous professional development competence* (*n* = 3), *Social and emotional competence* (*n* = 2) and *Content knowledge* (*n* = 2). The competencies included in the other four domains did not make the list of the 15 most important competencies.

## DISCUSSION

The aim of the study was to identify and describe the competencies that are essential for a HE teacher’s work. In the first round, 52 competencies were identified from the experts’ expressions (*n* = 343), and these were categorized into eight domains. In the second round, the expert panellists evaluated the importance of each competence and 40 most important were identified based on pre-defined cut-off values. In the third round, the experts ranked the 15 most important competencies from this set, and these competencies were divided into four fundamental domains.

### Fundamental HE teacher competence domains

The most important competencies were divided into four fundamental domains: *Pedagogic and subject-specific didactic competence, Social and emotional competence, Content knowledge* and *Continuous professional development competence*. A large proportion of the most important competencies belonged to the domain of *Pedagogic and subject-specific didactic competence*. This domain contained both theoretical and practical competencies, such as knowledge of the nature and specificities of HE as a school subject, curricula, teaching and learning processes, students and situation-specific skills. This domain is considered to be one of the fundamental domains for the teaching profession (Shulman, 1987), as it enables teachers to provide high-quality instruction (Voss *et al.*, 2011; Gess-Newsome *et al.*, 2017) and can foster students’ achievement (Baumert *et al.*, 2010).

*Content knowledge* also emerged as one of the fundamental domains of a HE teacher’s work. Good management of the subject content (i.e. facts, concepts, theories, phenomena) and mastery of the content structure helps in building logically progressive teaching entities. Studies have indicated a link between students’ learning and teachers’ content knowledge (Sadler *et al.*, 2013; Gess-Newsome *et al.*, 2017).

Without *Social and emotional competence*, the teacher may not be able to connect with students and shape the learning atmosphere in a manner that supports learning. Relational and emotional skills are required in daily classroom work (Jennings and Greenberg, 2009). These skills allow teachers to manage conflict situations and negotiate between differing viewpoints (Denham *et al.*, 2009; Jennings and Greenberg, 2009). They also support students in taking academic risks and seeking help from teachers (Newman, 2000). As noted above, neither *Pedagogic and subject-specific didactic competence* nor *Content knowledge* alone will lead to the best possible outcome. In HE, interaction with and between learners is important, since a shared understanding of many key concepts and phenomena already requires dialogue about their content and meaning. The personal, intimate, cultural and temporal nature of much of the content of HE, and also as the power relations inherent in learning situations, require the teacher to have high levels of *Pedagogic and subject-specific didactic competence, Social and emotional competence* and *Content knowledge*.

The complex nature of the teaching profession requires C*ontinuous professional development* to adapt to rapid changes, changing demands and evolving constraints or needs. The significance of continuous professional development in changing or developing teaching practices is widely accepted (van den Bergh *et al.*, 2015). Indeed, HE teachers should view themselves as learners during their teaching career, and a willingness to undertake higher-order thinking, reflection and self-regulation can make teachers better able to help students reach their full potential.

The overlap between fundamental domains poses a challenge for HE teacher training. Subject teacher education should avoid situations where domains and competencies are developed only in isolation or in individual courses; rather, the aim should be to create situations where student teachers can apply pedagogical and didactic competence, content knowledge and socio-emotional skills with real students in authentic situations. The fundamental domains are interconnected and develop reciprocally; hence opportunities for guided and reflective HE teaching practice are crucial.

### Supporting HE teacher competence domains

The other domains viewed as encompassing HE teachers’ core competencies were *Ethical competence, Competence in school health promotion, Contextual competence* and *Professional well-being competence.* Although none of these individual domains had competencies rated within the top 15 competencies, they were nevertheless indicated as being highly relevant to the work of a HE teacher (having high values of median and pairwise agreement, and low IQR of competencies).

The *Ethical competence* requirements of HE teachers are linked to their educational role and to their position of authority and expertise. Teachers’ personal values, and their commitment to ethical standards in teaching, guide their educational practices in everyday school life, influencing their ability to solve ethical problems and to act reasonably in morally loaded situations (Husu and Tirri, 2001). Professional values and ethical principles are indeed one of the key features of the teaching profession (Cambell, 2008). In HE, a teacher’s ethical competence is also linked to the objectives of the national curriculum, to the personalized and value-laden learning content and to the related pedagogical choices made. An understanding of the ethical nature of teaching guides the teacher in creating safe learning situations, which are essential when discussing difficult and sensitive issues. The development of students’ own ethical competence requires good ethical knowledge on the part of the teacher; the teacher can then build, preferably together with students, versatile learning situations that enable the student’s ethical competence to develop.

The primary task of the HE teacher is to teach the subject and support students in achieving the learning objectives of the curriculum. Nevertheless, *Competence in school health promotion* is a specific area of expertise for the HE teacher. The school and the teacher are subject to a wide range of expectations and responsibilities, and one such area is health promotion in schools, given that the school has been identified as an important arena for health promotion (Jourdan *et al.*, 2008). HE teachers therefore need to have a comprehensive understanding of both HE in the classroom (aiming to improve students’ health literacy) and health promotion in the school community as a whole (aiming to improve students’ health) (Mũkoma and Flisher, 2004). There is evidence that a health-promoting school (HPS) framework, applied holistically, can have a positive impact on some aspects of students’ health (Langford *et al.*, 2015).

HE teachers should have *Contextual competence*, i.e. a broad understanding of the socio-cultural environment of which the school is a part and where teaching takes place (Shulman, 1987). Local and national factors such as the structure of the neighbourhood, the socio-economic status of families, norms, values, the historical traditions of education and the subject and economic factors influence the conditions under which HE teachers work. Understanding the interrelation of these factors and critically reflecting on their impact contributes to educational equity (Darling-Hammond, 2006) and provides opportunities for both quality teaching and the reform of school culture to meet the challenges of the future.

*Professional well-being competence* is essential because the complexity of the teaching profession causes stress for many teachers. Extensive meta-analyses have shown that teacher stress and burnout are serious problems in many countries (García-Arroyo *et al.*, 2019; García-Carmona *et al.*, 2019). The stress experienced by teachers is negatively related not only to their work performance but also to their students. High levels of stress and low coping skills among teachers are associated with students’ behavioural problems and lower academic achievement (Herman *et al.*, 2018), and teachers’ emotional exhaustion has been found to be negatively related to students’ school satisfaction, grades and perceived teacher support (Arens and Morin, 2016). Hence, HE teacher training needs to address factors related to stress management, recovery and resilience.

Within teacher education, there are still perceptions that knowledge-based processing of issues and phenomena will enable trainees to deal successfully with practical situations and, moreover, the pedagogical methods used do not necessarily produce the desired learning outcomes at the level of teachers’ practical work (Korthagen, 2010; Cochran-Smith *et al.*, 2015). A broad and deep mastery of the relevant competencies requires appropriate pedagogical approaches in HE teacher education. This means that in developing teacher education, attention should be paid to the surface, deep and implicit structures of teaching practices (Shulman, 2005). In line with this, one has to ask several questions: do the teaching methods used in HE teacher education support student teachers’ performance in practical teaching situations? Do the teaching methods prepare them for unexpected situations and for decision-making under conditions of uncertainty? To what extent do student teachers encounter situations in their training where they have to question their assumptions, consider solution options or alternatives and analyse and justify their choices in the light of research and theoretical perspectives? How does teacher education guide students to consider the possibly hidden social and cultural norms or moral dimension of HE teacher work (consisting of, e.g. beliefs related to professional attitudes, values and dispositions)? Here it should be noted that a focus on the different structures of teaching practices (surface, deep and implicit) can help student teachers learn how to think and act within the work of the HE teacher, shaping their professional identity and mindset and supporting them in building professional values.

### Context-specific versus universal competencies

The cultural, social and task-specific context of teaching influences the competencies considered essential for teaching (Berliner, 2001). Some teacher attributes are universal, while others may be specific to a particular educational environment (Klassen *et al.*, 2018). This study was carried out in Finland, which has distinct features concerning schools and teachers. Overall, teachers in Finland are considered to be trusted professionals. Research-based teacher education gives teachers the ability to work independently; they also have a high degree of pedagogical autonomy and can apply the curriculum as they see fit (Sahlberg, 2010).

The Finnish context offers an exceptional opportunity to study the competencies required of HE teachers. Finland is one of the few countries in the world where HE is an independent and obligatory school subject in comprehensive and secondary education (Paakkari and Paakkari, 2019). The situation of HE as a compulsory subject in schools for over 20 years has accumulated a wide range of views on the competencies required for HE teachers. There are clearly defined eligibility criteria for HE teachers (a master’s degree, including at least 60 credits in HE studies and 60 credits in pedagogy). Over several decades, work has been done on the theoretical basis of the subject and on teacher education programmes, the national curricula have been revised several times, and teaching methods, textbooks and other teaching materials have been actively developed. In addition, a national assessment of learning outcomes in HE has been carried out (Summanen, 2014). These factors provide a unique perspective on the role of HE in schools and on the competencies needed to conduct effective teaching.

While these contextual factors are important, it should be noted that the research-based understanding of education has become more unified worldwide (Paine *et al.*, 2016). Overall, the research literature emphasizes a learner-oriented and constructivist approach, the active role of students in learning and the teacher’s support for a diverse student body. These factors are independent of country, educational environment or tradition (Paine *et al.*, 2016). They require similar competencies from teachers, and thus the results of this study have international implications.

### Teacher competencies and the development of health literacy

The main goal of HE, i.e. the development of health literacy, differs greatly from that of other school subjects. Health literacy is something that can be learned and developed. Because schools reach almost the entire age group at any given time, competent HE teachers have an excellent opportunity to develop health literacy in children and adolescents in an equitable ways and to reduce avoidable disparities in health literacy, due (for example) to socio-economic status (IUHPE, 2018; Paakkari *et al.*, 2019a, 2019b). However, this requires adequate resources (e.g. access to education, well worked-out school curricula, sufficient lessons), plus high-quality university-level teacher education to develop teachers’ competencies comprehensively and systematically. Health literacy includes several dimensions. These include an adequate knowledge base on health issues, health-related skills (e.g. searching for information, using digital services), the ability to think critically (e.g. assessing the reliability of information and health communication, critically evaluating health determinants), the ability to act in an ethically sustainable way (e.g. the ability to take social responsibility and act in ways that promote one’s own health and the health of others, and the ability to consider the probable consequences of one’s actions on others) and, in addition, understanding of one’s own needs, perceptions and wishes in relation to health and well-being (Paakkari and Paakkari, 2012; World Health Organization, 2021). Teacher training should therefore develop HE teachers’ understanding of the key features and nature of health literacy, as well as promote the competencies described above to help teachers develop students’ health literacy levels. This will enable them to prepare students to face the health challenges of current and future society, which include, for example, managing infodemics, detecting health-related mis- and disinformation, and understanding the potential and problematic nature of artificial intelligence.

### Limitations and further research

This study has some limitations. The goal behind the experts’ selection in this study was to increase qualitative strength in defining core competencies in the HE teachers’ profession. However, a different selection of panellists could have produced different or additional core competencies. The distinctive features of Finland as a HE context should be understood, and caution is needed in generalizing the results globally. The teaching profession is complex and requires a variety of competencies. In the survey, panellists were forced to select the 15 most important competencies for HE teachers. This highlights the core competencies, but may give a narrow view of the overall competencies and fundamental domains required for a HE teacher’s work.

Identifying and defining the core competencies of the HE teacher opens up opportunities for further research. The identified competencies could form the basis of an instrument to measure the level and development of HE student teachers’ competencies during their training and thus assess the effectiveness of teacher education. Such an instrument could also be used to study the development of teachers’ competencies at different stages of their careers. Data collected at different points in time could be used to develop teacher training in HE. It would also be interesting to compare HE teacher education in different countries. In this way, one could discover the competencies that are emphasized, possible strengths or weaknesses, and success in developing particular competencies. An international comparative study would also provide opportunities to assess the comprehensiveness of the model created in this study. This would indicate whether there are certain country-specific characteristics that should be taken into account when defining competencies. It would also be interesting to further investigate the relationship between teacher competencies and student learning outcomes in HE.

## CONCLUSION

The core of the HE teacher profession is broad expertise beyond teacher knowledge. Building competence-based curricula into HE teacher education requires the identification of core HE competencies. Clearly defined domains and competencies can increase conceptual coherence and reduce the risk of fragmented teacher education programmes (Grossman *et al.*, 2009). The core competencies can be used to evaluate the content, emphasis, prioritization needs and future directions of HE teacher education programmes. Defined teacher competencies can serve as a development and assessment tool for self-evaluation and reflection among pre- and in-service HE teachers and teacher trainees.

Defining individual competencies and domains is important for the development of teacher education, but it can give a simplistic picture of the expertise required of a HE teacher. The domains, and the competencies they contain, form a broad set with considerable overlap, and a HE teacher’s proficiency in a particular domain does not guarantee quality teaching or optimal student learning. It is therefore essential to understand the relationships between the competence domains, plus the opportunities and constraints they create for the HE teacher’s work, and to take account of this interconnectedness when developing HE teacher education.
